# Supplementary material for: Is It Time to Commit to a Process to Re-Evaluate Oncology Drugs? A Descriptive Analysis of Systemic Therapies for Solid Tumour Indications Reviewed in Canada from 2017 to 2021
Source: Curr Oncol. 2022 Mar 10;29(3):1919–31. doi: 10.3390/curroncol29030156 (PMC8947363; doi:10.3390/curroncol29030156)
Supplement: Supplementary file 1 [file curroncol-29-00156-s001.zip › curroncol-1539283-supplementary.pdf]

Table S1. List of all drugs for solid tumour indications approved by Health Canada from January 1, 2017 to October 31, 2021.

Generic drug name	Standard, Priority, or Accelerated NOC/c review	Year of Health Canada Approval	Pivotal Trial Design	Indication	ESMO MCBS Score	CADTH Recommendation
Atezolizumab	Standard	2021	RCT	NSCLC (1L metastatic) (PD-L1)	5	No submission
Brigatinib	Standard	2021	RCT	NSCLC (ALK+)(1L)	3	Positive
Encorafenib + binimetinib	Standard	2021	RCT	Metastatic melanoma (BRAF)	4	Positive
Encorafenib + cetuximab	Standard	2021	RCT	Colorectal cancer (BRAF)	Not available	Positive
Infigratinib phosphate	NOC/c	2021	Phase II	Bile duct FGFR2+ (previously treated)	3	No submission
Lorlatinib (monotherapy)	Priority	2021	RCT	NSCLC ALK+ (1L)	4	Positive (draft)
Lurbinectedin	NOC/c	2021	Phase II	Small cell lung cancer (previously treated)	2	No submission
Neratinib + capecitabine	Standard	2021	RCT	Metastatic Breast cancer (HER2)	1	No submission
Nivolumab	Priority	2021	RCT	adjuvant esophageal cancer	A	Ongoing
Nivolumab + ipilimumab	Standard	2021	RCT	malignant pleural mesothelioma	3	Positive
Nivolumab + ipilimumab	NOC/c	2021	Phase II	Colorectal cancer (MSI-HI)	3	No submission
Osimertinib	Priority	2021	RCT	NSCLC (adjuvant)	A	Positive (draft)
Pembrolizumab + chemo	Priority	2021	RCT	esophageal cancer (HER2-)	Not available	Positive (draft)
Pemigatinib	Standard	2021	Phase II	Bile duct FGFR2+ (previously treated)	3	Negative (draft)
Pertuzumab, trastuzumab	Standard	2021	RCT	Neoadjuvant EBC (HER2+)	C	Negative (draft)
Pralsetinib	NOC/c	2021	Phase II	RET+ NSCLC	3	No submission
Selpercatinib	NOC/c	2021	Phase II	RET+ NSCLC, thyroid	3	Ongoing
Sotorasib	NOC/c	2021	Phase II	NSCLC KRAS+ (previously treated)	Not available	No submission
Tepotinib	NOC/c	2021	Phase II	NSCLC (MET+)	3	Ongoing
Trastuzumab deruxtecan	NOC/c	2021	Phase II	Breast cancer	2	No submission
Alpelisib	Standard	2020	RCT	Breast cancer (PIK3CA, HER2-)	3	Negative (draft)
Atezolizumab + bevacizumab	Standard	2020	RCT	Non-squamous NSCLC	3	Negative
Atezolizumab + bevacizumab	Priority	2020	RCT	hepatocellular carcinoma	5	Positive
Avelumab	Priority	2020	RCT	urothelial carcinoma (maintenance post 1L)	4	Positive

Durvalumab	Standard	2020	RCT	Extensive-stage small cell lung cancer	3	Positive
Entrectinib	Standard	2020	Phase II)	NSCLC (ROS-1)	3	Positive
Entrectinib	NOC/c	2020	Phase II	NTRK fusion (tumour agnostic)	3	No submission
Niraparib (monotherapy)	Priority	2020	RCT	Ovarian cancer (1L)	3	Positive
Nivolumab + ipilimumab	Standard	2020	RCT	NSCLC (PD-L1)	2	No submission
Nivolumab + ipilimumab + chemo	Priority	2020	RCT	NSCLC (1L)	4	Positive
Olaparib	Priority	2020	RCT	pancreas	3	No submission
Olaparib (monotherapy)	Priority	2020	RCT	mCRPC	3	Positive
Pembrolizumab	Standard	2020	RCT	Head & neck (PD-L1)	4	Positive
Pembrolizumab	NOC/c	2020	Phase II	bladder cancer	3	No submission
Pembrolizumab	Standard	2020	RCT	NSCLC (PD-L1 > 1%)	5	No submission
Ribociclib + AI	Standard	2020	RCT	Breast (HR+, HER2-) (pre-menopausal)	5	Positive
Ribociclib + fulvestrant	Standard	2020	RCT	Breast (HR+, HER2-)	4	Positive
Ripretinib	Priority	2020	RCT	GIST	3	Ongoing
Sonidegib	Standard	2020	phase II (RCT)	Basel cell carcinoma	Not available	Negative
Tucatinib + trastuzumab + capecitabine	Priority	2020	phase II (RCT)	Breast cancer (HER2+)	3	Positive
Abemaciclib	Standard	2019	RCT	Breast cancer (HER2-)	3 (AI), 4 (FUL)	Positive
Atezolizumab + chemo	Priority	2019	RCT	Extensive-stage small cell lung cancer	3	Negative
Atezolizumab + chemo	NOC/c	2019	RCT	Triple negative breast cancer (PD-L1)	3	Withdrawn
Cabozantinib	Standard	2019	RCT	Hepatocellular carcinoma	3	Positive
Cemiplimab	NOC/c	2019	Phase II	Adv Cutaneous Squamous Cell Carcinoma	3	Positive
Dacomitinib	Standard	2019	RCT	NSCLC EGFR+	3	Positive
Erdafitinib	NOC/c	2019	Phase II	Urothelial cancer (FGFR)	1	No submission
Larotrectinib	NOC/c	2019	Phase II	NTRK fusion (tumour agnostic)	3	Positive
Lorlatinib (monotherapy)	NOC/c	2019	Phase II	NSCLC ALK+ (post crizotinib + another ALKi)	3	Negative

Lutetium (177Lu) oxodotretotide	Priority	2019	RCT	Neuroendocrine tumours	4	Positive
Neratinib (monotherapy)	Standard	2019	RCT	Extended Adjuvant Breast cancer (HER2)	A	Negative
Niraparib (monotherapy)	Standard	2019	RCT	Ovarian cancer (maintenance post-chemo)	3	Positive
Olaparib	Priority	2019	RCT	maintenance for 1L ovarian cancer	4	Positive
Pembrolizumab	Standard	2019	RCT	Adjuvant melanoma	A	Positive
Pembrolizumab	Standard	2019	Phase II	urothelial carcinoma (PD-L1)	3	Negative
Pembrolizumab	NOC/c	2019	RCT	MSI HI endometrial or colorectal cancer	4	Positive (CRC)
Pembrolizumab + axitinib	Standard	2019	RCT	Renal cell carcinoma	4	Positive
Pembrolizumab + chemo	Standard	2019	RCT	Squamous NSCLC	4	Positive
Pembrolizumab + lenvatinib	NOC/c	2019	Phase II	Endometrial cancer (not MSI HI)	3	Ongoing
Pembrolizumab + pemetrexed + chemo	Standard	2019	RCT	NSCLC (non-squamous) (1L)	4	Positive
Talazoparib	Standard	2019	RCT	Breast cancer (HER2-)	4	No submission
Tipiracil hydrochloride, trifluridine	Priority	2019	RCT	Gastic cancer	3	Positive
Trastuzumab emtansine	Priority	2019	RCT	Adjuvant breast cancer (HER2+)	A	Positive
Alectinib	Priority	2018	RCT	NSCLC (ALK+)(1L)	4	Positive
Atezolizumab	Standard	2018	RCT	NSCLC (2nd line)	5	Positive
Avelumab	NOC/c	2018	Phase II	Urothelial carcinoma (post chemo)	Not available	No submission
Bevacizumab	Standard	2018	RCT	Glioblastoma	Not available	No submission
Brigatinib	NOC/c	2018	phase II	NSCLC (ALK+) (progressed)	3	Negative
Cabozantinib	Standard	2018	RCT	Renal cell carcinoma	3	Positive
Dabrafenib + trametinib	Priority	2018	RCT	Adjuvant melanoma (BRAF)	A	Positive
Dabrafenib + trametinib	Priority	2018	Phase II	NSCLC BRAF	2	Positive
Dinutuximab	Standard	2018	RCT	Neuroblastoma	A	Positive
Durvalumab	NOC/c	2018	RCT	NSCLC (maintenance post chemo)	4	Positive
Lenvatinib	Standard	2018	RCT	hepatocellular carcinoma	Not available	Positive
Nivolumab	NOC/c	2018	Phase II	hepatocellular carcinoma	Not available	Negative
Nivolumab	Standard	2018	RCT	adjuvant melanoma	A	Positive

Nivolumab + ipilimumab	Priority	2018	RCT	Renal cell carcinoma	4	Positive
Olaparib (monotherapy)	Priority	2018	RCT	breast (BRCA+) (previously treated)	4	No submission
Osimertinib	Priority	2018	RCT	NSCLC (1L)(EGFR+)	4	Positive
Pertuzumab + trastuzumab	Standard	2018	RCT	Breast (HER2+) EBC	A	Negative
Ribociclib + AI	Standard	2018	RCT	Breast (HR+, HER2-) (post-menopausal)	3	Positive
Tipiracil hydrochloride, trifluridine	Priority	2018	RCT	Colorectal cancer	3	Negative
Atezolizumab	NOC/c	2017	Phase II	urothelial carcinoma	3	No submission
Avelumab	NOC/c	2017	Phase II	Merkel Cell Carcinoma (progression)	3	Positive
Crizotinib	Standard	2017	Phase II	NSCLC (ROS-1)	3	Positive
Durvalumab	NOC/c	2017	Phase II	Urothelial cancer (after progression)	Not available	No submission
Eribulin mesylate	Standard	2017	RCT	Sarcoma	3	No submission
Erlotinib hydrochloride	Standard	2017	RCT	NSCLC (EGFR) (maintenance post chemo)	1	No submission
Lenvatinib + everolimus	Standard	2017	phase II (RCT)	Renal cell carcinoma (progressed)	4	Negative
Necitumumab + chemo	Standard	2017	RCT	NSCLC	1	No submission
Nivolumab	Priority	2017	RCT	Head & neck	5	Positive
Olaratumab	NOC/c	2017	phase II (RCT)	Soft tissue sarcoma	Not available	Positive
Palbociclib + fulvestrant	Standard	2017	RCT	Breast (HR+, HER2-)	4	Positive
Panitumumab	Standard	2017	RCT	Colorectal cancer (wild type RAS)	3	Negative
Pembrolizumab (monotherapy)	Priority	2017	RCT	urothelial carcinoma (post chemo)	4	Positive
Regorafenib	Priority	2017	RCT	hepatocellular carcinoma (progressed)	4	Positive

Abbreviations: ESMO-MCBS, European Society of Medical Oncology-Magnitude of Clinical Benefit Scale; GI, gastrointestinal; GU, genitourinary; NOC, Notice of Compliance; NOC/c, Notice of Compliance with conditions; NSCLC, non-small cell lung cancer; RCT, randomized controlled trial
